# Cloning and characterization of norbelladine synthase catalyzing the first committed reaction in Amaryllidaceae alkaloid biosynthesis

**DOI:** 10.1186/s12870-018-1570-4

**Published:** 2018-12-07

**Authors:** Aparna Singh, Marie-Ange Massicotte, Ariane Garand, Laurence Tousignant, Vincent Ouellette, Gervais Bérubé, Isabel Desgagné-Penix

**Affiliations:** 1 Department of Chemistry, Biochemistry and Physics, Université du Québec à Trois-Rivières, 3351 boul. des Forges, Trois-Rivières, QC G9A 5H7 Canada; 2 Plant Biology Research Group, Université du Québec à Trois-Rivières, 3351 boul. des Forges, Trois-Rivières, QC G9A 5H7 Canada

**Keywords:** Amaryllidaceae alkaloid, Pathogenesis related protein 10, Alkaloid biosynthesis, *Narcissus pseudonarcissus*, Norcoclaurine synthase, Norbelladine

## Abstract

**Background:**

Amaryllidaceae alkaloids (AAs) are a large group of plant-specialized metabolites displaying an array of biological and pharmacological properties. Previous investigations on AA biosynthesis have revealed that all AAs share a common precursor, norbelladine, presumably synthesized by an enzyme catalyzing a Mannich reaction involving the condensation of tyramine and 3,4-dihydroxybenzaldehyde. Similar reactions have been reported. Specifically, norcoclaurine synthase (NCS) which catalyzes the condensation of dopamine and 4-hydroxyphenylacetaldehyde as the first step in benzylisoquinoline alkaloid biosynthesis.

**Results:**

With the availability of wild daffodil (*Narcissus pseudonarcissus*) database, a transcriptome-mining search was performed for *NCS* orthologs. A candidate gene sequence was identified and named *norbelladine synthase* (*NBS*). *NpNBS* encodes for a small protein of 19 kDa with an anticipated pI of 5.5. Phylogenetic analysis showed that *Np*NBS belongs to a unique clade of PR10/Bet v1 proteins and shared 41% amino acid identity to opium poppy NCS1. Expression of *NpNBS* cDNA in *Escherichia coli* produced a recombinant enzyme able to condense tyramine and 3,4-DHBA into norbelladine as determined by high-resolution tandem mass spectrometry.

**Conclusions:**

Here, we describe a novel enzyme catalyzing the first committed step of AA biosynthesis, which will facilitate the establishment of metabolic engineering and synthetic biology platforms for the production of AAs.

**Electronic supplementary material:**

The online version of this article (10.1186/s12870-018-1570-4) contains supplementary material, which is available to authorized users.

## Background

The Amaryllidaceae alkaloids (AAs) are a group of naturally synthesized molecules with more than 600 renowned complex structures [1]. They are pharmacologically active compounds that are classified under three different groups of C-C phenol coupling namely *para-para’*, *ortho-para’* and *para-ortho’* [2]. An outsized variety of pharmacologically active AAs have been identified with the bioactive properties including the acetylcholine esterase inhibitor galanthamine, anti-tumor activity of lycorine and the cytotoxic haemanthamine [3–5]. AAs are obtained chiefly from the extracts of plants from *Galanthus, Leucojum* and *Narcissus* species, as their complicated structures do not enable cost-effective high-yield organic synthesis [6]. Though AAs display a large range of pharmaceutical applications, only galanthamine is accessible in markets as an Alzheimer’s treatment drug because of its ability to stabilize behavioral symptoms in the course of six months treatment in comparison to chemically synthesized acetylcholinesterase inhibiting drugs, donepezil and rivastigmine [7].

**Fig. 1:**
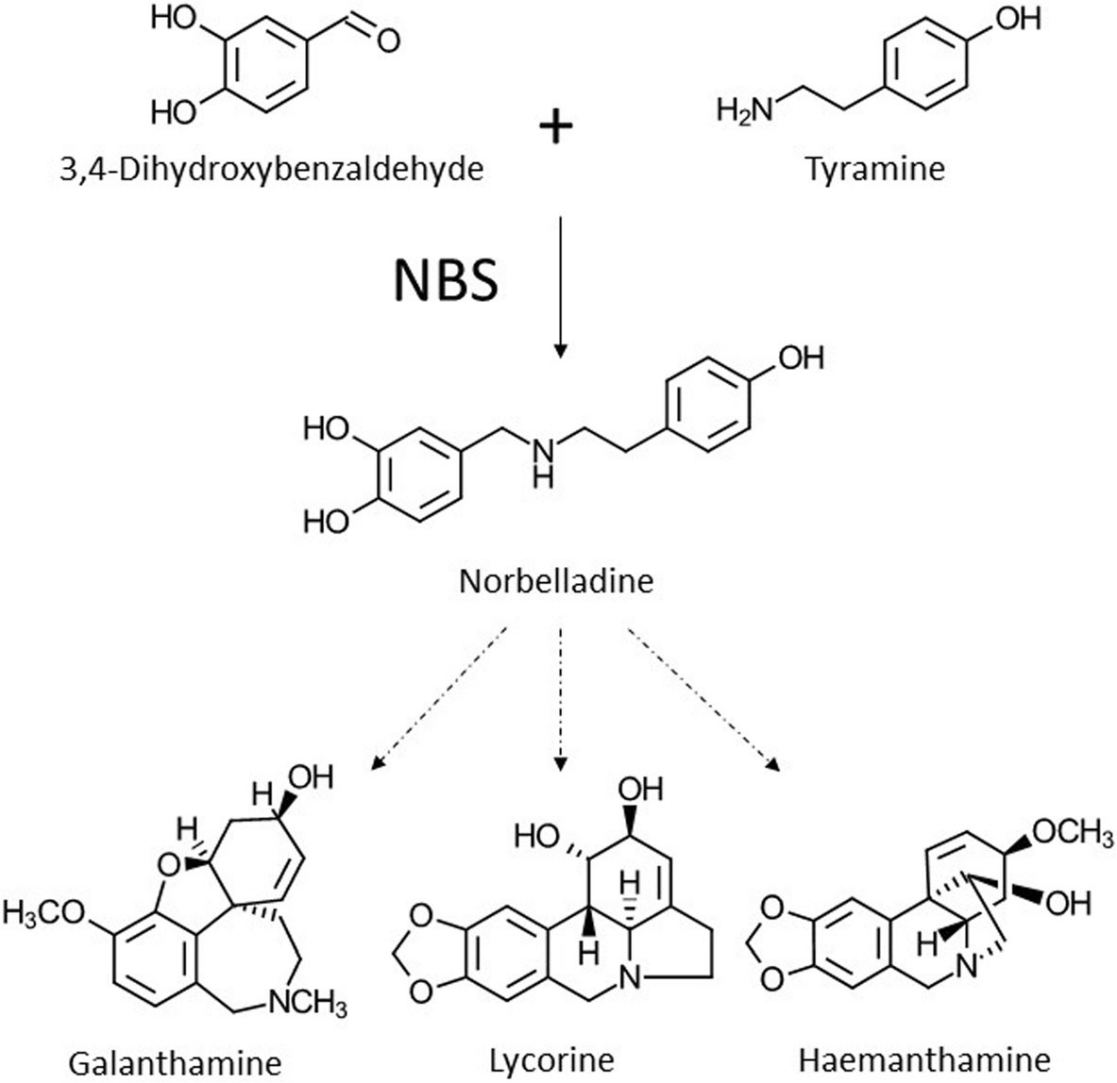
Norbelladine synthase (NBS) catalyzes the condensation of tyramine and 3,4-dihydroxybenzaldehyde (3,4-DHBA) to form norbelladine, the common precursor to all Amaryllidaceae alkaloids produced in plants including galanthamine, lycorine and haemanthamine

Previous investigations on the biosynthesis of AAs *in planta* have revealed that all AAs are made from the common metabolic intermediate, norbelladine (Fig. 1) [8–14]. For example, radiolabeled studies showed that deuterium labelled 4’-*O-*methylnorbelladine was incorporated in all three different groups of AAs [11]. To date, only three AAs biosynthetic genes have been identified including the *norbelladine 4’-O-methyltransferases* (*N4OMT*), encoding N4OMT enzyme catalyzing norbelladine methylation at 4′ position to form 4’-*O*-methylnorbelladine [15], *CYP96T1*, encoding a cytochrome P450 enzyme which catalyzes the synthesis of (*S,R*)-noroxomaritidine [16] and *noroxomaritidine reductase* (*NR*) encoding the enzyme that catalyzes the formation of oxomaritinamine [17]. However, to this date, the enzyme catalyzing the first committed step leading to norbelladine synthesis has not been identified.

It has been proposed that norbelladine is formed by the condensation of tyramine and 3,4-dihydroxybenzaldehyde (3,4-DHBA) via a Mannich reaction [18]. The involvement of the Mannich reaction has been proposed in many biosynthetic pathways, especially for alkaloids. For example, norcoclaurine synthase (NCS), catalyzing the first committed step in benzylisoquinoline alkaloids formation, condenses the two precursors molecules; dopamine and 4-hydroxyphenylacetaldehyde to form norcoclaurine [19]. Since the AA biosynthetic pathway shares analogy with the benzylisoquinoline alkaloid biosynthesis, we proposed that a *NCS* ortholog in Amaryllidaceae may be a candidate that is capable of catalyzing the formation of norbelladine.

Hence for this study, we applied BLAST searches for candidate sequences orthologous to *NCS* in our previously generated de novo transcriptome from wild daffodil (*Narcissus pseudonarcissus*) [20]. The *NCS* orthologous sequence identified named *norbelladine synthase* (*NBS*) was isolated, cloned in an expression vector and transformed into *E. coli.* Following heterologous expression and purification, NBS recombinant protein was used for enzyme assay and LC-MS/MS analysis of the product showed that NBS has the ability to convert 3,4-DHBA and tyramine to norbelladine. Thus, we report on the first characterization of norbelladine synthase, the enzyme which catalyzes the first committed step in AAs biosynthesis.

## Results

### Candidate gene identification

RNA from *N. pseudonarcissus* ‘King Alfred’ bulb was isolated and corresponding cDNA was used for Illumina sequencing, which provided a deep transcriptome database to search for expressed genes encoding enzymes involved in AA biosynthesis [20]. To identify orthologous sequence to *NCS* in *N. pseudonarcissus* transcriptome, we performed BLASTx sequence similarity searches with *NCS* from *Thalictrum flavum, Papaver somniferum*, *Argenome mexicana, Eschscholzia californica, Chelidonium majus and Coptis japonica*. Three resulting orthologous full-length transcripts were obtained (TR17354|c0_g1_i1, TR17354|c0_g1_i2 and TR17354|c0_g1_i3) consisting of 593, 575 and 583 bp with corresponding open reading frames of 492, 480 and 483 bp, respectively. Their E-value to *NCS* ranges between 1e^− 20^ to 4e^− 20^. The three transcripts were 97% identical, though small differences were observed towards the 3’end (Additional file 1). The candidate displaying the longest predicted ORF of 492 bp was named *N. pseudonarcissus* *Norbelladine* *Synthase* (*NpNBS)* and was used for subsequent functional analysis. The ORF encoded protein consisted of 163 amino acids with an anticipated molecular weight of 19 kDa and a theoretical pI of 5.5. BLAST and motif scan analyses revealed the presence of conserved Bet v1 and Pathogenesis-Related (PR-10) protein domain. The PR-10/Bet v1 allergen proteins do not possess NCS activity although they share similarity to NCS protein. Thus, *Np*NBS, similarly to NCS, belongs to the PR-10/Bet v1 protein family.

### Phylogenetic analysis

**Fig. 2:**
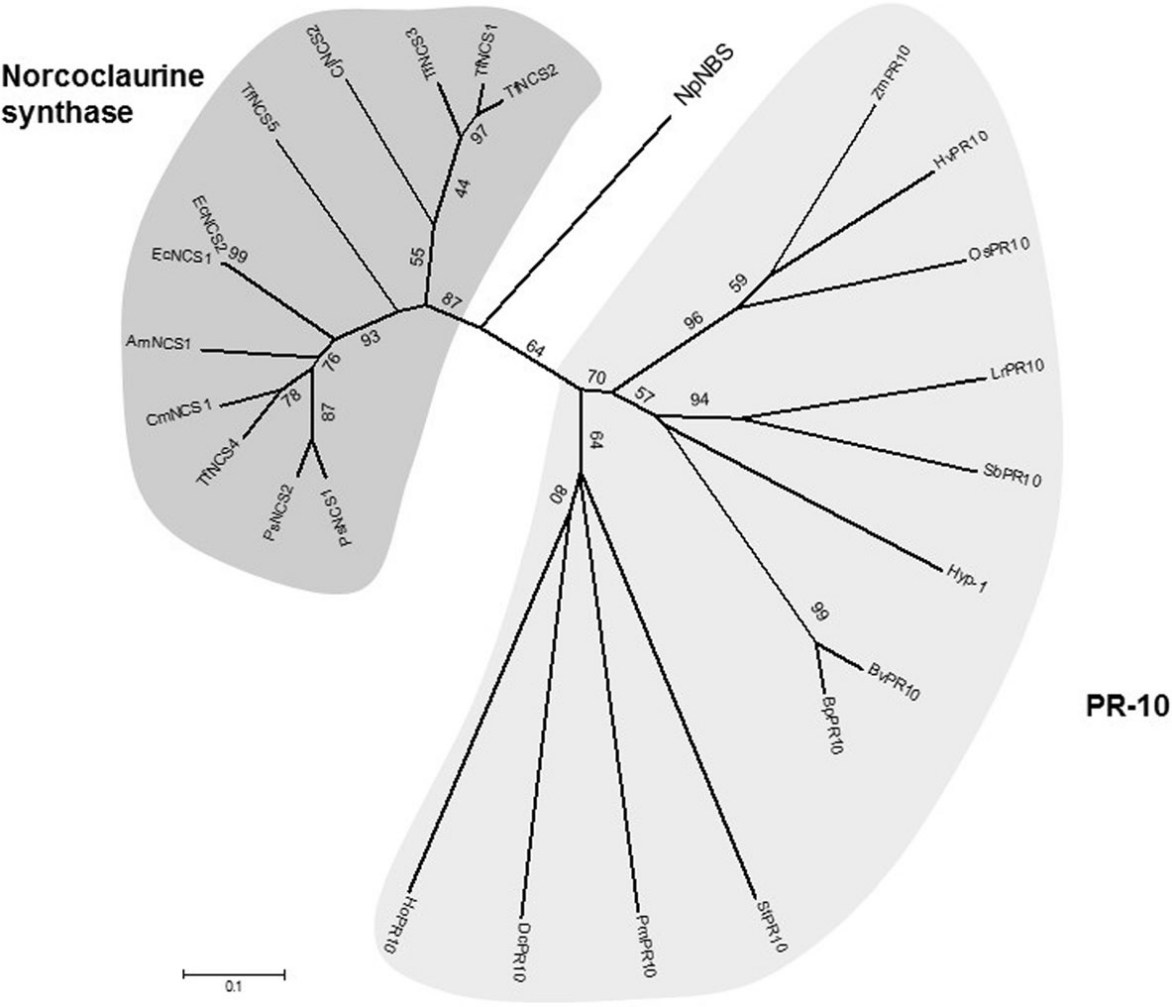
Phylogenetic relationships among several PR10/Bet v 1 proteins from a variety of plants. Phylogeny tree was based on the amino acid sequences for *Betula verrucosa BvPR10*, *Betula platyphylla BpPR10, Papaver somniferum Ps*NCS, *Ps*NCS2, *Thalictrum flavum TfNCS*, *TfNCS2*,*TfNCS3*, *TfNCS4*, *TfNCS5*, *Chelidonium majus CmNCS1*, *Argenome mexicana AmNCS*, *Eschscholzia californica EcNCS1*, *EcNCS2, Coptis japonica CjNCS2 (formerly CjPR10A), Daucus carota DcPR10*, *Solanum tuberosum StPR10*, *Hyacinthus orientalis HoPR10, Pinus monticola PmPR10, Hordeum vulgare HvPR10, Oryza sativa OsPR10, Lily regale LrPR10, Sorghum bicolor SbPR10, Zea mays ZmPR10* and *Hypericum perforatum Hyp-1.* Accession numbers provided in Additional file 2. The evolutionary history was inferred using the Neighbor-Joining method [36] with bootstrap value 500. The optimal tree with the sum of branch length = 5.78745187 is shown. The tree is drawn to scale, with branch lengths in the same units as those of the evolutionary distances used to infer the phylogenetic tree. The evolutionary distances were computed using the p-distance method [43] and are in the units of the number of amino acid differences per site. The analysis involved 25 amino acid sequences. All positions containing gaps and missing data were eliminated. There were a total of 139 positions in the final dataset. Bootstrap values were added to the figure. Evolutionary analyses were conducted in MEGA 6 [38]

To investigate the evolutionary history of *Np*NBS and its relationship with PR-10/Bet v1 proteins, a phylogenetic tree was constructed with the predicted amino acid sequence of *Np*NBS. Although all proteins are members of the PR-10 protein family, the phylogenetic tree was clearly divided into two main clusters: the PR-10 and the Norcoclaurine synthases (Fig. 2; Additional file 2). This type of clustering was reported previously [21]. Notably, *Np*NBS shares higher homology with proteins from the NBS cluster including 38% amino acid sequence identity with *Thalictrum flavum Tf*NCS1/2 and 41% with *Papaver somniferum Ps*NCS1/2. *Np*NBS displayed comparatively lower identity to proteins of the PR-10 cluster including 19% identity to *Betula verrucosa Bv*PR10 and only 7% to *Hyacinthus orientalis Ho*PR10. Although *Np*NBS sequence is located the edge of the PR-10/NCS cluster, it is more closely related with the NCS group since it showed higher homology with *Tf*NCS (Fig. 2). Altogether the results suggest that NBS, NCS and PR-10 evolved as distinctive genes over time.

**Fig. 3:**
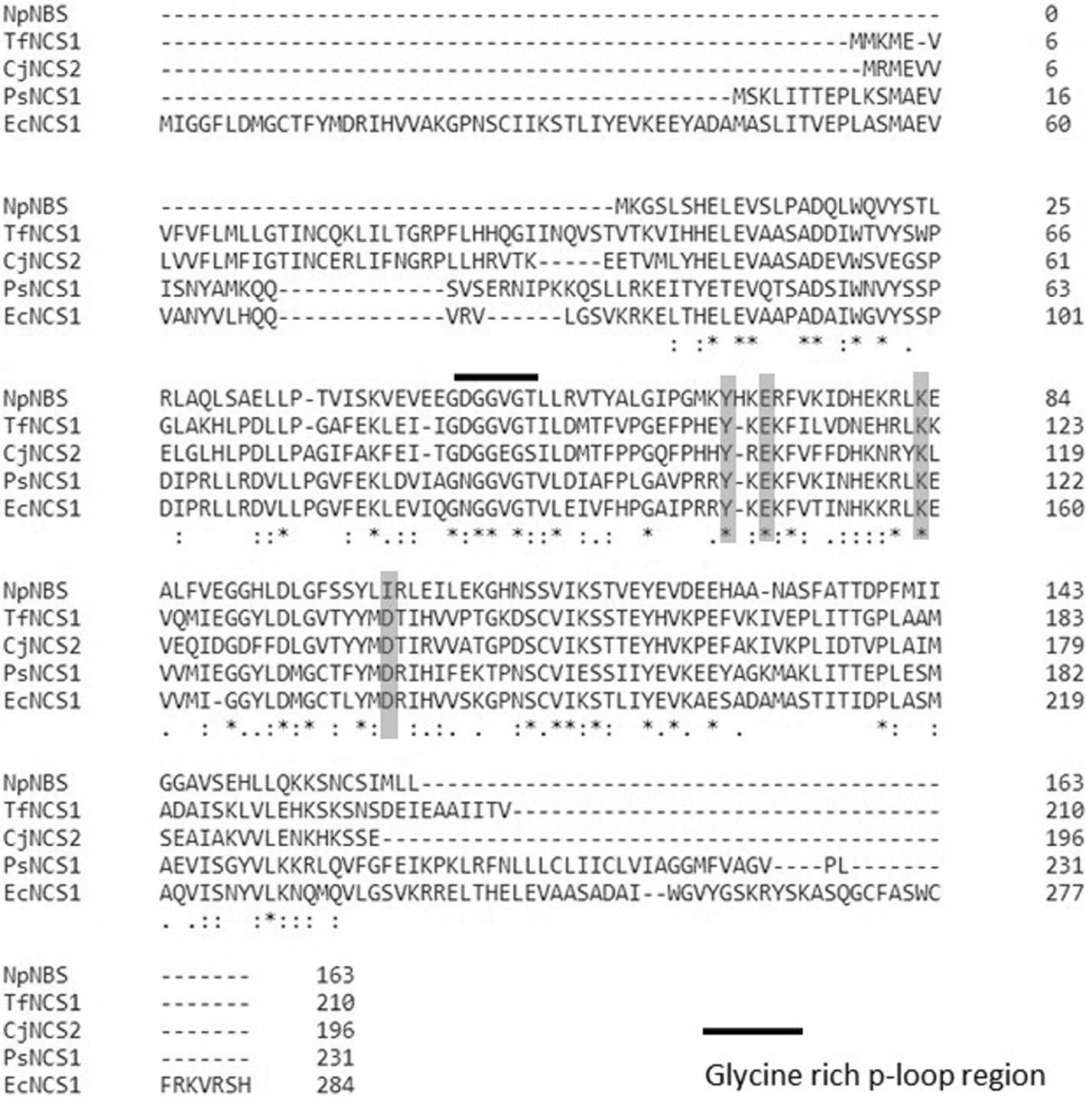
Clustal omega alignment of deduced amino acid sequence of *Narcissus pseudonarcissus* norbelladine synthase (*Np*NBS) with *norcoclaurine synthase* (NCS) amino acid sequences obtained from different species: *Eschscholzia californica Ec*NCS1, *Thalictrum flavum Tf*NCS1, *Papaver somniferum Ps*NCS1, and *Coptis japonica Cj*NCS2 (formely *Cj*PR10A). Grey boxes corresponding to Tyr, Lys, Asp and Glu residues forms the *NpNBS* catalytic residue. The black bar depicts a glycine-rich P-loop domain conserved in *NCS* and *NpNBS* protein. The fully conserved residues are marked with an asterisks (*). The positions with conservation between amino acid residues of similar properties are marked with a colon (:) and the positions with conservations between amino acids residues of weakly similar properties are marked with a period (.). Numbers on the right indicate the residues position in the sequence

To identify regions of similarity that may be important for defining functional and structural relationships, multiple sequences alignment were performed using *NpNBS* and the NCS amino acid sequences from various plant species (Fig. 3). Thus, *Ec*NCS1, *Ps*NCS1, *Cj*NCS2 (formely CjPR10A) and *Tf*NCS1 genes shared over 38% identity in amino acid sequences with each other, while they showed the same identity (38%) with *Np*NBS. The alignment also showed the presence of NCS conserved catalytic residues Tyr^108^, Glu^110^, Lys^122^ and Asp^141^ (Fig. 3). The residues were renamed as Tyr^68^, Glu^71^, Lys^83^, according to their position in *NpNBS* protein sequence*.* However, the NCS Asp^141^ was not found in *Np*NBS sequence and was replaced by an isoleucine residue. All amino acid sequences contained a phosphate-binding loop (P-loop) glycine-rich region (Fig. 3). The glycine-rich loop is the conserved ligand-binding domain of Bet v1 protein family [22]. Subcellular localization of the *Np*NBS amino acid sequence was predicted using several programs, including SignalP4.1 and WoLF PSORT, and the result indicated the absence of signal peptides. In addition, localization predictor program (PSLpred) identified *Np*NBS as a cytosolic protein. Altogether, the data suggest that *Np*NBS proteins are localized in cytoplasm.

### RT-qPCR analysis

**Fig. 4:**
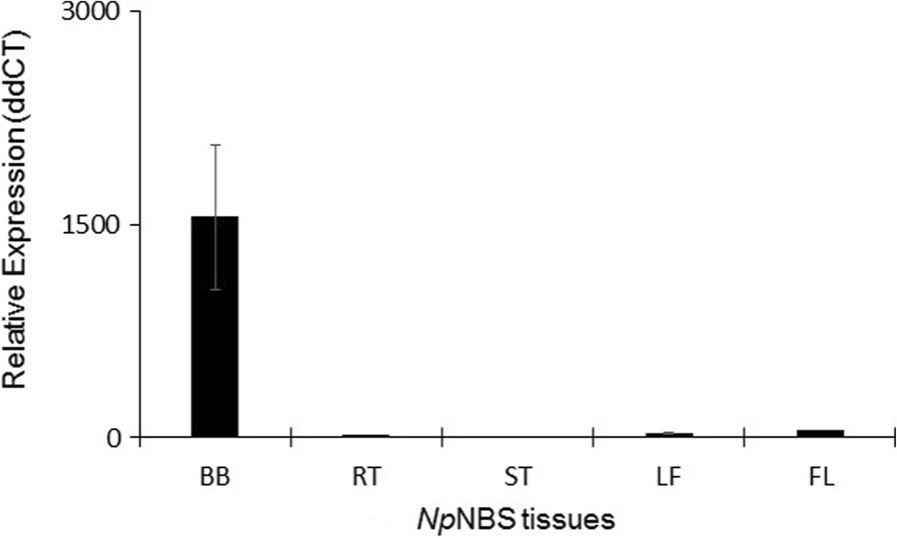
Reverse transcription quantitative PCR analysis of *NpNBS* expression in different tissues of *N. pseudonarcissus* ‘King Alfred’. Plant tissues were harvest after flowering in spring. Graph is plotted using normalized ddCT values scaled to lowest ddCq value by dividing ddCq of a sample with a minimum ddCq value identified among the samples [39]. Histone was used for internal reference. Expression fold change were calculated using the comparative 2^-ΔΔCt^ method from three independent replicates. Bars represent the mean standard deviation of three independent replicates. Abbreviations: tissues are BB, bulb; RT, root; ST, stem; LF, leaf; FL, flower

The study of *NpNBS* expression profile in different tissues of *N. pseudonarcissus* ‘King Alfred’ by reverse transcription quantitative PCR (RT-qPCR) revealed high expression of *NpNBS* in bulbs compared to roots, stems, leaves and flowers (Fig. 4). The normalized ddCT expression of *NpNBS* in other tissues was detected below 60, whereas its expression in bulbs was approximately 1500 folds higher. High expression of *NpNBS* relates to the higher AAs content in bulbs of *N. pseudonarcisssus* ‘King Alfred’ [20].

### *Np*NBS gene cloning and heterologous expression

**Fig. 5:**
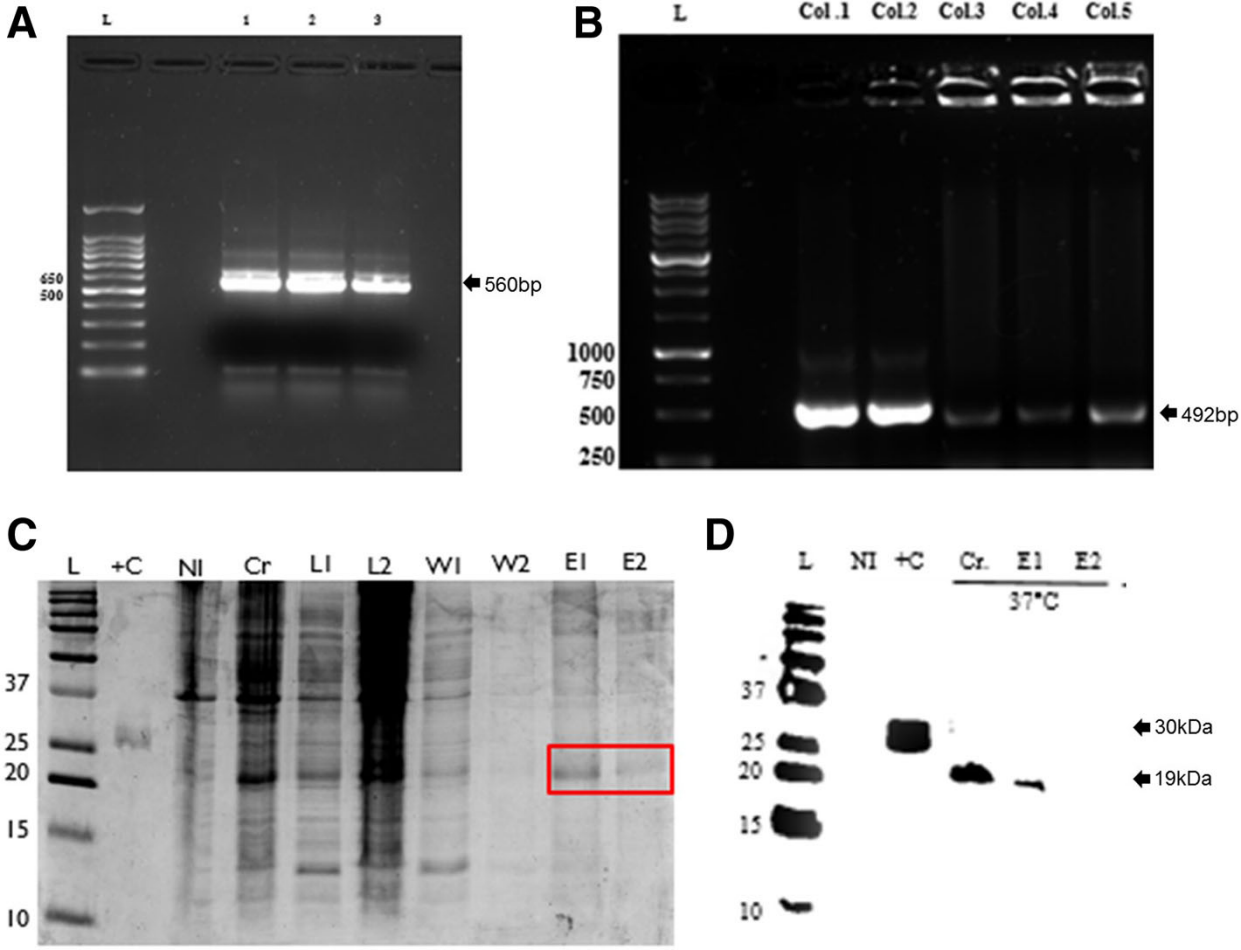
Heterologous expression of *Np*NBS in *Escherichia coli*. **a** Agarose gel electrophoresis of PCR amplified candidate *Np*NBS from a *N. pseudonarcissus* cDNA library. Product size is 560 bp (ORF 492 bp + Gateway adapters 68 bp). Numbers on the left refer to the location of standard DNA molecular markers in bp. **b** Agarose gel electrophoresis of PCR amplified *Np*NBS performed with 5 positively transformed bacterial colonies (col.1 to col.5) shows a band of *Np*NBS ORF transcript size of 492 bp, L is for Ladder; **c** SDS-PAGE analysis of *Np*NBS protein. Purified protein was extracted from 0.9 mM IPTG induced *E. coli* Rosetta™ (DE3) pLysS host strain at 37 °C for 8 h in elution 1 with 300 mM of imidazole. L-Ladder; +C- positive control with a 30 kDa protein; NI-non-induced protein; L1- lysate 1 (10 mM imidazole); L2- lysate 2 (10 mM imidazole); W1-wash 1 (20 mM imidazole); W2-wash 2 (20 mM imidazole); E1-elution 1 (300 mM imidazole); E2-elution 2 (300 mM imidazole). **d** Western blot analysis performed using 6x HIS-tag epitope antibody shows expression of recombinant protein at 37 °C in 0.9 mM IPTG induced crude and pure protein extracts. No detectable expression of recombinant protein at 25 °C in 0.9 mM IPTG induced crude and pure protein extracts. Lane L-ladder; NI- protein non-induced; +C-positive control protein with C-terminal His tags 30 kDa; Cr-crude protein 37 °C/8 h/0.9 mM; E1- elution 1, 37 °C/8 h/0.9 mM; E2- elution 2, 37 °C/8 h/0.9 mM; Cr-crude 25 °C/8 h/0.9 mM; E1- elution 1, 25 °C/8 h/0.9 mM; E2-elution 2, 25 °C/8 h/0.9 mM. Numbers on the left refer to the location of standard protein molecular weight markers in kDa

To produce recombinant *Np*NBS, a full-length *NpNBS* cDNA was PCR amplified from *N. pseudonarcissus*. The amplified product of *NpNBS* shows a band of 566 bp on the 1% agarose electrophoresis gel (Fig. 5a). *NpNBS* was gel purified, cloned into the pET301 expression vector with a C-terminal 6x-His tag, transformed into *E. coli* competent cells and the positive colonies were selected on LB supplemented with ampicillin and chloramphenicol. Colony PCR confirmed the presence of positive colonies (Fig. 5b) which were induced with IPTG at 37 °C and protein fractions were extracted. SDS-PAGE analysis was performed with different fractions obtained during *Np*NBS protein purification process which show bands of different sizes, suggesting the presence of numerous bacterial cell proteins in fractions from the crude (Cr), lysate (L1 and L2) and wash buffer (W1 and W2). An apparent molecular mass of 19 kDa in elution buffer 1 (E1) was observed, which was absent in non-induced protein (NI) (Fig. 5c). Western blot analysis was performed using 6x His-tag monoclonal antibody. A His-tag *Np*NBS crude (Cr) and purified extracts (E1) isolated from transformed bacterial cell culture grown at 37 °C showed the expected protein at molecular weight of 19 kDa. No protein expression was observed in cultures without IPTG (NI) (Fig. 5d). The results showed successful production of recombinant *Np*NBS from IPTG-induced bacterial cell culture.

### *Np*NBS enzyme assay

For the biosynthesis of all AAs, the precursor norbelladine is made from a the condensation of tyramine and 3,4-DHBA which forms the imine norcraugsodine followed by its reduction to norbelladine [17]. It is not clear if norbelladine formation is one stepwise reaction catalyzed by a single enzyme or two separate reactions (condensation and reduction) catalyzed by different enzymes. To confirm *Np*NBS protein function, enzyme assays were carried out with purified *Np*NBS recombinant protein. The resulting assay product was subjected to LC-MS analysis using a Positive Electrospray Ionization mode (+ESI). The QqQ dual +ESI source conditions were optimized using freshly synthesized alkaloid standards, norbelladine and norcraugsodine (Additional file 3) to obtain a high sensitivity. The MS/MS parameters such as capillary voltage, spray voltage and skimmer voltage were enhanced to maximize the ionization in the source and sensitivity to identify and characterize all possible fragmentation products. With standards, we observed predicted major mass spectral fragments for the nobelladine *m/z* 260 [M + H]^+^ and norcraugsodine *m/z* 258 [M + H] ^+^, both at 5.5 min (Additional file 3). Fragmentation of norbelladine molecular ion *m/z* 260 [M + H]^+^ yielded ion fragments of *m/z* 159, 138, 123 and 121 (Additional file 3). The ion fragment *m/z* 138 was obtained by the elimination of the 4-ethylphenol moiety (122 Da) whereas *m/z* 121 was produced by loss of tyramine. Precisely for norbelladine, the transition [M + H]^+^ of 260 → 138 was selected for the qualifier ion fragment and the transition [M + H]^+^ of 260 → 121 was used as the quantifier ion (Additional file 3). Similar MS/MS ion fragments spectra were reported for norbelladine suggesting a good fragmentation of our standard [17].

**Fig. 6:**
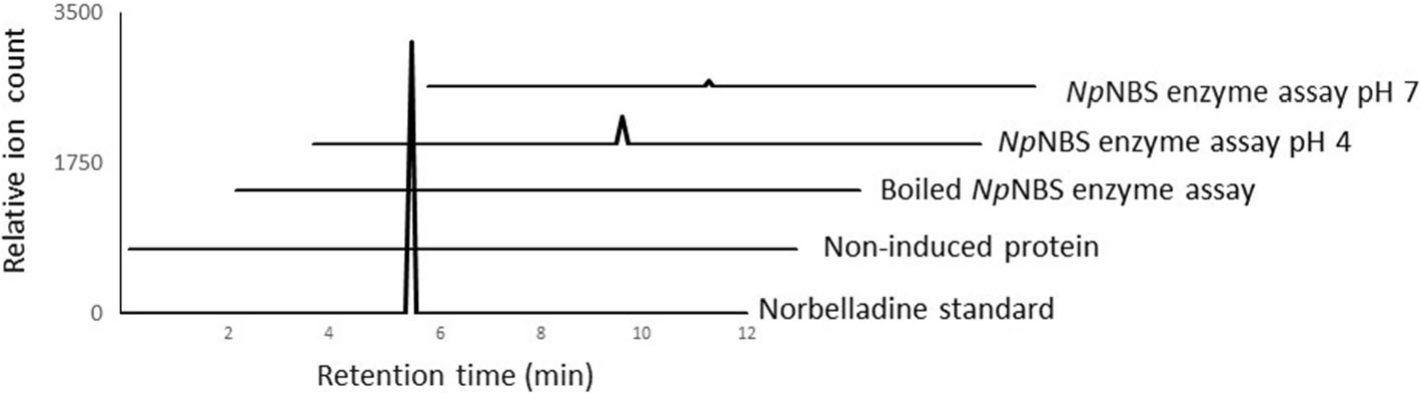
Extracted ion chromatograms showing the product of *Np*NBS enzyme assays. The tested substrates used were 3,4-dihydroxybenzaldehyde (300 μM) and tyramine (10 μM), the extracted ion chromatogram corresponds to standard NB or assays conducted with the non-induced protein fraction, the heat-denatured recombinant *Np*NBS enzyme; and the complete assay performed with recombinant *Np*NBS at pH 4 and pH 7. Parent ion mass-to-charge (*m/z*) of 260 for norbelladine and 258 for norcraugsodine were subjected to collision-induced dissociation analysis for identification and quantitation (Additional file 3)

Enzyme assays with *Np*NBS yielded a peak at 5.5 min on LC-MS which was the same retention time as those of the norbelladine and norcraugsodine standards. The +ESI-MS/MS analysis of the *Np*NBS enzyme assay product showed the presence of the qualifier and quantifier ion fragments of norbelladine (Fig. 6). The level of product was detected in the *Np*NBS assay pH 4 and at lower levels at pH 7. Norcraugsodine molecular ion and fragment ions were not detected in the assay. It should be noted that a very low background reaction was observed in control assay/non-induced protein assay (i.e. when the two substrates were in contact without NpNBS) suggesting a low level of non-enzymatic condensation. However, these levels were 1000 times lower than those measured in presence of *Np*NBS. *Np*NBS enzyme assay product shows that *Np*NBS catalyzes the condensation reaction between tyramine and 3,4 DHBA to produce norbelladine. Thus, the in vitro enzyme assay demonstrated ability of norbelladine synthase activity of *Np*NBS with a preference at pH 4.

## Discussion

The formation of norbelladine is a crucial step in the formation of Amaryllidaceae alkaloids. In this study, we identified the enzyme norbelladine synthase in wild daffodil (*N. pseudonarcissus*) responsible for the condensation of tyramine with 3,4-dihydroxybenzaldehyde (3,4-DHBA) to form norbelladine. *Np*NBS contains conserved Bet v1 domain and thus is a member of the Bet v1/PR10 protein family. The PR-10 family universally exist in plants of monocots and dicots group [23–25]. Apart from the PR-10 family, recently Bet v1 have shown an extended low level similarity with three more protein families; major latex and ripening-related proteins, norcoclaurine synthase proteins, and cytokinin-binding proteins from legumes [26]. Several studies have also demonstrated that members of the Bet v1/PR-10, specifically of the NCS group, have a crucial role in alkaloid production [27, 28]. For example, *Ps*NCS from opium poppy shares significant amino acid identity with PR-10 and Bet v1 protein and NCS has a major role in benzylisoquinoline alkaloid biosynthesis. Indeed, Virus-Induced Gene Silencing (VIGS) of *PsNCS1* and *PsNCS2* transcripts resulted in a 75 to 82% decrease of main alkaloids in opium poppy latex compared to control [29].

NCS catalyzes the condensation of dopamine with 4-hydroxyphenylacetaldehyde to yield (*S*)-norcoclaurine, the central precursor of benzylisoquinoline alkaloids. *Np*NBS was identified from transcriptome data search using *NCS* sequence homology. The high level of *NpNBS* expression in bulbs correlates with elevated content (and diversity) of AAs compared to other plant parts suggesting the importance of *Np*NBS in AA metabolism. Interestingly, UPLC-QTOF-MS detected 4-hydroxybenzaldehyde, the pathway precursor to 3,4-DHBA the co-substrate for *Np*NBS, only in underground tissues such as bulb and roots [20]. *Np*NBS shared 41% amino acid sequence identity with *P. somniferum* NCS1 and NCS2 but lacked the signal peptide that could be involved in subcellular localization. Furthermore, motif searches predicted that *Np*NBS is a cytosolic protein and is not associated with a subcellular compartment such as endoplasmic reticulum, mitochondria or peroxisomes. The observed catalytic activity of *Np*NBS confirmed its role in AA biosynthesis. The shorter N-terminal region of *Np*NBS, 41 amino acid less compared to *Tf*NCS (Fig. 3), and the absence of the signal peptide in the sequence did not affect its activity suggesting that it is not required. These results are consistent with studies conducted on *Tf*NCS which reported that a signal peptide had no role in the catalytic activity of an enzyme. Samanani et al. (2004) [19] studied the activity of *Tf*NCS protein encoding truncation of first 10 and 29 N-terminal amino acids which resulted in formation of (*S*)-norcoclaurine. Both recombinant proteins displayed enzymatic activity [19]. In addition, a study conducted on four N-terminally truncated variants of *Cj*NCS of 10, 19, 29 and 42 amino acids residues revealed an increasing fold of enzymatic activity. For example, *Cj*NCS-Δ19 displayed 10-fold (0.30 U/mL) and *Cj*NCS-Δ29 had shown 40-fold (1.20 U/mL) higher activity compared to full length *Cj*NCS (0.03 U/mL) [29]. Recently, Li et al. (2016) reported on the isolation and characterization of NCS variants from several alkaloid-producing plant species. They found that in some case, NCS orthologs possess two, three or four repeated catalytic domains and their presence was found to be proportional with increase in catalytic efficiency [30].

*Np*NBS enzyme catalyzes norbelladine synthesis by a condensation process between amine and aldehyde. Among the proposed catalytic residues, the Glu^71^ was detected with + 1 histidine shift in *Np*NBS which might affect the interaction with catalytic and hinder the cyclization process. Moreover, *Np*NBS abilty to form norbelladine in the absence of Asp^141^ catalytic residues suggests that Asp^141^ residue is not responsible for basic catalysis and might be involved in imparting electrostatic stabilization [31]. The *Np*NBS enzyme displays higher activity at pH 4 indicating that the reaction may prefer an acidic environment. Similarly, pH of 6.2 has been reported for optimum acitivity of *Ps*NCS [32].

The high expression of *NpNBS* in bulbs is supported by its high FPKM value obtained in the transcriptome [20]. *NpNBS* was reported among the top expressed genes, comprising 16.12% of the *N. pseudonarcissus* ‘King Alfred’ transcriptome. *NpNBS* high expression was consistent with the expression of *NpN4OMT* in bulbs, which supported the abundant occurrence of AAs in bulb indicating that bulb is an important site for enzyme localization and AAs biosynthesis [20]. Moreover, Kilgore et al. 2014, also presented a similar correlation between high expression and read count of *N4OMT* with abundant galanthamine accumulation in bulb tissues [15]. Therefore, based on the expression data, we conclude that *Np*NBS has a crucial role in AAs metabolism. A similar high *Tf*NCS expression and enzymatic activity was reported in underground rhizome and root tissues compared to other parts, suggesting tissue-specific localization of NCS enzyme [33].

## Conclusions

The rapid progress in the discovery of AAs biosynthesis pathway genes along with newly characterized *NpNBS* enzyme is useful for reconstituting a short synthetic AAs biosynthesis pathway in yeasts or microbes that will allow feasible and high-scale production of targeted medicinally important Amaryllidaceae alkaloids which are gaining interest for their new pharmaceutical applications, including an Alzheimer’s treatment drug, galanthamine [33, 34]. The applied strategy could be implemented in characterization of genes from any class of specialized metabolites as our discovery shows the utility of the designed workflow.

Although the origin of alkaloid biosynthesis is not always clear, the key genes involved in these pathway are gradually being isolated. For example, norcoclaurine synthase couples dopamine and 4-hydroxyphenylacetaldehyde as the first committed step in benzylisoquinoline alkaloid biosynthesis [19, 21]. Another example is strictosidine synthase, which catalyzes the first specific step in monoterpenoid indole alkaloid biosynthesis [35]. *Np*NBS could offer a new perspective for studies on the diversity and evolution of alkaloid biosynthesis. The condensation of tyramine and 3,4-DHBA is central to the biosynthesis of myriad AAs in plants. We demonstrated that the enzyme responsible for this reaction is a member of the PR10/Bet v1 family of proteins. Further work on *Np*NBS will include attempts to determine the optimum pH and temperature and to probe the scope of alternate substrates in the reaction. The use of this enzyme in biotechnological microbial platforms will provide an attractive alternative to the production of AAs, including several pharmacologically important compounds such as galanthamine and lycorine.

## Methods

### Plant tissue and chemicals

Wild daffodil (*Narcissus pseudonarcissus* ‘King Alfred’) bulbs were purchased from Fraser’s Thimble farms (BC, Canada). Bulbs were planted in the field in Trois-Rivieres (Québec, Canada) during the month of October and were not harvested until flowering stage in early May. Different tissues such as bulbs, roots, stems, leaves and flowers were collected separately, flash frozen in liquid nitrogen and stored at − 80 °C. Ampicillin, HPLC grade acetonitrile, agarose, methanol, 6x His-tag epitope tag antibody and monosodium phosphate were purchased from Fisher Scientific (Janssen Pharmaceuticalaan, Geel, Belgium). 3, 4-dihydroxybenzaldehyde (3,4-DHBA) were obtained from Acros organic (New Jersey, USA). Tyramine and chloramphenicol were bought from Sigma-Aldrich (MO, USA). *Taq* DNA polymerase was purchased from gene direx. Sodium chloride (NaCl), isopropyl β-D-1-thiogalactopyranoside (IPTG), and imidazole were bought from Fisher Bioreagents/scientific (New Jersey, USA). The SensiFAST SYBER Lo-Rox kit for reverse transcription-quantitative PCR was obtained from Bioline (London, U.K). The Gateway cloning kit was purchased from Invitrogen (CA, USA). Ni-NTA his-tag affinity columns were purchased from Qiagen (Germany), plasmid miniprep kit acquired from Geneaid (Taipei, Taiwan). Mini-protean TGX stain-free precast gels, 4x-Laemmli buffer, 10–250 kD precision plus kaleidoscope prestained protein standard ladder and clarity western ECL substrate were was obtained from Bio-rad (USA) and kanamycin was purchased from Bioshop (Burlington, ON, Canada).

### RNA extraction, Illumina sequencing and transcriptome assembly

The transcriptome was generated in a previously published study by Singh and Desgagné-Penix, 2017 [20]. Briefly, total RNA from bulbs of *N. pseudonarcissus* ‘King Alfred’ was extracted using the Cetyltrimethylammonium bromide method, converted to cDNA and sequenced using Illumina HiSeq 2000 PE. The raw pair reads were trimmed, cleaned, normalized, and assembled into a transcriptome [20]. Of 73,081,603 raw paired reads, a total of 10,523,999 surviving paired reads after normalization were assembled into 195,347 transcripts. The sequences were deposited in the National Center for Biotechnology Information Sequence Read Archive (https://www.ncbi.nlm.nih.gov/sra/?term=SRR5788585) under the accession number SRR5788585.

### Candidate gene identification

The sequence for norbelladine synthase was deposited to GenBank and the GenBank accession number for the nucleotide sequence is MG948545.

### Phylogenetic tree and protein alignment

Sequences listed in Additional file 2 were aligned using CLUSTAL W in MEGA 6 software with default parameters. The evolutionary history was inferred using the Neighbor-Joining method using the branch lengths contained in the inferred tree [36]. Divergence times for all branching points in the topology were calculated with the RelTime method [37]. Phylogenetic analysis was conducted on MEGA 6 [38]. Protein sequence of *NpNBS* was aligned with norcoclaurine synthase sequences from *Coptis japonica* (*CjNCS2), Papaver somniferum (PsNCS1), Eschscholzia californica (EcNCS1) and Thalictrum flavum* (*TfNCS1)* using Clustal omega. *NpNBS* sequence was analyzed for signal peptide using Signal-BLAST (http://sigpep.services.came.sbg.ac.at/signalblast.html) and PSLpred (http://crdd.osdd.net/raghava/pslpred). Motif scan search for *NpNBS, PsNCS1, BtPR10* and *CjNCS* sequences was performed using myhits (http://myhits.isb-sib.ch/cgi-bin/motif_scan).

### Reverse transcription-quantitative PCR (RT-qPCR)

*N. pseudonarcissus* cDNA for bulbs, roots, stems, leaves, and flowers was generated from 1 to 2 μg of RNA using the Qiagen omniscript RT kit according to manufacturer’s protocol (QIAGEN, Germany). The experiment was performed in triplicate. A total reaction volume of 20 μL containing 1x SensiFAST SYBR Lo-ROX mix, 200 μM of each forward and reverse primers (Additional file 4) and cDNA sample was used for RT-qPCR analysis. Histone was used as internal reference gene. Real-time quantitative PCR was performed on CFX Connect RT-qPCR System from Bio-rad (USA). PCR conditions for amplification were 95 °C for 3 mins, 95 °C for 10 s, annealing temperature 52 °C for 30 s for 40 cycles. This was followed by dissociation step (as provided by software) - 95 °C for 10 s, 65 °C for 5 s and 95 °C for 5 s. The amplification efficiency was determined at 92% and a melting curve analysis confirmed *NpNBS* PCR product specificity. Norbelladine synthase relative expression values were determined by comparative 2^-ΔΔCt^ method and were scaled to lowest ddCq value by dividing ddCq of a sample with a minimum ddCq value identified among the samples [39].

### PCR and cloning

The open reading frame (ORF) of full length *NpNBS* was amplified from *N. pseudonarcissus* bulbs cDNA with 200 μM dNTPs, 1.25 unit *Taq* DNA polymerase in 50 μL reaction and 0.2 μM forward and reverse gateway primers (Additional file 4). PCR program parameters: 3 mins 94° 1 cycle, 30 s 94°, 45 s 52°, 1 min 72° for 30 cycles, 5 min 72 °C 1 cycle. A Gateway cloning technology was used to clone *NpNBS* according to manufacturer’s protocol. The gateway-adapted *att*P-flanked pDONR 221 vector with kanamycin resistance gene was used for BP recombinase reaction (catalyzed by BP clonase enzyme) to generate an *att*L-flanked entry clones with *att*B-flanked *NpNBS* DNA fragment. These entry clones were transformed into *E. coli* DH10β competent cells and positive clones were obtained on a kanamycin selection plate. Chloramphenicol was used for counterselection of positive clones. These clones were further used to perform a LR recombination reaction between an *att*L-containing entry clone and *att*R-containing pET301/CT-DEST destination vector (150 ng/μL) with a histidine tags and T7 promoter, lac operator and *attB* recombination site while C-terminal contain 6x His-tag, T7 reverse priming site and T7 terminator. Positively transformed *E. coli* DH10β competent cells were selected from ampicillin-Luria-Bertani (LB) media plates incubated overnight at 37 °C. The positive colonies were obtained using 100 μg/mL ampicillin and confirmed by sequencing.

### Protein expression

The above extracted plasmid were transformed into *E. coli* Rosetta™ (DE3) pLysS host strain for protein expression using the heat shock transformation protocol [40]. Transformed cells were placed onto Luria-Bertani (LB) media with ampicillin and chloramphenicol selection plates overnight at 37 °C. A single colony was picked and grown overnight at 37 °C at 200 rpm in 7 mL LB broth containing ampicillin (100 μg/mL) and chloramphenicol (34 μg/mL). The overnight grown pre-culture was diluted 1:100 in fresh LB broth containing ampicillin (100 μg/mL) and chloramphenicol (34 μg/mL) and grown at 200 rpm at 37 °C to an A_600nm_ between 0.5–0.8. Isopropyl-β-D-thiogalactopyranoside (IPTG) was added to a final concentration of 0.9 mM to induce protein expression. The culture was incubated for 8 h at 37 °C at 200 rpm. The supernatant and pellet of the IPTG-induced bacterial culture were separated by centrifugation at 9032 x *g* for 15 min and stored at − 80 °C. *E. coli* crude cell pellet were used to purify *Np*NBS protein. Non-induced protein (not treated with IPTG) was also collected from bacterial pellet.

### Protein purification and Western blotting

Protein purification was performed by resuspending cell pellet, obtained from IPTG induced *E. coli* Rosetta (DE3)pLys cell cultures, in 10 mg/mL lysozyme and lysis buffer containing 50 mM NaH_2_PO_4_, 300 mM NaCl and 10 mM imidazole pH 8 and incubated on ice for 30 min. After sonication and centrifugation at 10,000 x *g* for 20 min at 4 °C, supernatant containing the 6x His-tagged protein was collected (Lysate 1), which was loaded on pre-equilibrated nickel affinity spin columns from Qiagen (Germany) and centrifuged at 900 x *g* for 5 min. Clear lysate (Lysate 2) was collected and saved for SDS-PAGE analysis. Nickel affinity columns were washed twice with 600 μL wash buffer containing 50 mM sodium phosphate monobasic (NaH_2_PO_4_), 300 mM sodium chloride (NaCl) and 20 mM imidazole pH 8 by centrifugation at 900 x *g* for 2 min and flow thorough were saved as wash 1 and wash 2 for SDS-PAGEanalysis. At last, protein was eluted twice (elution 1 and 2) each time in 300 μL elution buffer with 50 mM NaH_2_PO_4_, 300 mM NaCl and 300 mM imidazole pH 8 by centrifugation at 900 x *g* for 2 min and eluate was collected. Protein quantification was done according to Bradford assay [41]. Five different BSA concentrations (5,10,15, 20, 25 and 50 μg) were prepared and absorbance (mean of three) was determined at 595 nm. A graph was plotted with above BSA concentrations at x-axis and their absorbances at y-axis respectively, which gave a linear equation of standard curve (y = 0.112x + 0.0357) and extinction coefficient (R^2^ = 0.999). The NBS protein concentration (x) was calculated using above equation by replacing y with NBS absorbance recorded at 595 nm. Protein was resolved on 15% Mini-protean TGX stain-free precast gels. Gels were transferred on nitrocellulose membrane, equilibrated with TBS buffer (20 mM Tris, 150 mM NaCl pH 7.5) for 15 min on a rotatory shaker, followed by blocking of membrane for 1 h. with Tris-buffered saline (TBS) containing tween 20 (TBST) and 1% bovine serum albumin (BSA). Nitrocellulose membrane was incubated overnight at 4 °C in TBST with 1% BSA containing 6x-His epitope tag antibody in 1:1000 dilution. After primary antibody incubation, membrane washed five times each for five minutes in TBST buffer and incubated for 1 h. in TBST containing 2.5% dry milk and goat anti-mouse horse radish peroxidase (GAM)-HRP conjugate in 1: 20,000 dilution. Immunoblot was washed six times for 5 min each in TBST buffer and developed using clarity Western ECL substrate from Bio-rad (USA).

### Nuclear magnetic resonance spectroscopy and mass spectrometry

Proton and carbon NMR spectra were recorded on a Varian 200 MHz NMR apparatus. Chemicals shifts (*δ*) are recorded in parts per million (ppm). Coupling constant are expressed in Hz. Samples were dissolved in DMSO-d_6_ for data acquisition (*δ* 2.49 ppm for ^1^H NMR and 39.95 ppm for ^13^C NMR) using TMS as internal standard (*δ* 0.00 ppm). Multiplicities were described by the following abbreviations: s for singlet, d for doublet, dd for doublet of doublets, t for triplet and m for multiplet. Mass spectral data was obtained from NanoQAM (Université du Québec à Montréal) using a Time-of-Flight LC/MS (LC/MS-TOF), Agilent Technologie, LC 1200 Series/6210 TOF-LCMS with electrospray ionization and positive mode (ESI+).

### Norbelladine synthesis

Norbelladine was synthesized using a previously published protocol with modifications [42]. A summary of the method is described below along with the result of the NMR spectral data (Additional file 5).

#### Step A: Synthesis of the imine norcraugsodine

An equimolar quantity of 3,4-dihydroxybenzaldehyde (251.6 mg, 1.82 mmol) and tyramine (249.8 mg, 1.82 mmol) were added as powders to a flask containing dichloromethane (7 mL). The solution was stirred gently for 6 h at room temperature to give the imine intermediate. The solvent was evaporated under reduced pressure with a rotatory evaporator and then, with a mechanical pump to remove solvent residue and water. The product obtained was sufficiently pure to be used as such in the next step. Crude yield, 99%; yellow solid.

^1^H NMR (200 MHz, DMSO-d_6_) *δ*: 7.98 (1H, s, CH imine), 7.15 (1H, d, J = 2.0 Hz, CH-Ar), 6.99 (2 H, d, J = 8.6 Hz, 2 x CH-Ar), 6.90 (1 H, dd, J_1_ = 2 Hz and J_2_ = 8.2 Hz, CH-Ar), 6.65 (1 H, d, J = 8.6 Hz, CH-Ar), 6.63 (2 H, d, J = 8.6 Hz, 2 x CH-Ar), 3.63 (2H, t, J = 7.2 Hz, CH=NCH_2_CH_2_), 2.73 (2H, t, J = 7.4 Hz, CH=NCH_2_CH_2_); ^13^C NMR (200 MHz, DMSO-d_6_) *δ*: 160.85, 155.90, 149.49, 146.08, 130.46, 130.12, 127.76, 121.88, 115.77, 115.42, 113.85, 62.41, 36.76; ESI+ HRMS: (M + H)^+^calculated for C_15_H_16_NO_3_ = 258.1125; found = 258.1078.

#### Step B: Synthesis of norbelladine

The imine norcraugsodine (50.6 mg, 0.98 mmol) was dissolved in methanol (5 mL) and was hydrogenated to the amine norbelladine using 30 mol% palladium on carbon (Pd/C 10%) under a H_2_ atmosphere. The hydrogen was bubbled three times (*t* = 0, 30 and 60 min) during the hydrogenation process. The mixture is agitated for a total of 2 h and then filtered on a silica gel to remove the Pd/C and impurities. Methanol was evaporated under reduced pressure with a rotatory evaporator and then, with a mechanical pump to give norbelladine. The product is a brownish solid. Yield: 98%.

^1^H NMR (200 MHz, DMSO-d_6_) *δ*: 6.94 (2 H, d, J = 8.6 Hz, CH-Ar), 6.63 (4 H, m, 4 x CH-Ar), 6.52 (1H, dd, J_1_ = 1.7 Hz and J_2_ = 7.7 Hz, CH-Ar), 3.49 (2H, s, Ar-CH_2_-NH), 2.58 (4 H, m, NH-CH_2_CH_2_-Ar); ^13^C NMR (200 MHz, DMSO-d_6_) *δ*: 155.8, 145.4, 144.3, 132.1, 130.9, 129.8, 119.2, 116.0, 115.6, 115.5, 53.1, 51.1, 35.4; ESI+ HRMS: (M + H)^+^ calculated for C_15_H_18_NO_3_ = 260.1281; found = 260.1182.

##### Enzyme assays

The screening assays contained 10 μg of purified protein, 10 μM tyramine, 300 μM 3,4-DHBA, 100 mM Tris buffer in a total volume of 90 μL. The assay was incubated at 37 °C for 2 h followed by termination using 3 μL of 20% trichloroacetic acid (TCA). Negative control includes non-induced proteins from *E. coli* (0.0 mM IPTG) whereas negative control assays include purified *Np*NBS protein boiled at 95 °C for 15 min.

The analysis of the enzymatic product of NBS was performed on a Waters 2690 high performance liquid chromatograph coupled to a Micromass Quattro LC mass spectrometer using a Kinetex C18 column (150 mm long × 4,6 mm inside diameter, 5 μM particle size). Samples were subjected to positive-mode electrospray ionization (ESI[+]) liquid chromatography [27]-tandem mass spectrometry (MS/MS) for reaction product characterization, including collision-induced dissociation (CID) fragmentation analysis. Ten microlitres of each sample was injected onto the column and compounds were eluted at a flow rate of 0.25 mL/min using ammonium acetate 10 mM, pH 5.0 (solvent A) and acetonitrile 100% (solvent B). The LC program started with 40% solvent B, a gradient began at 0 min to 98% at 7 min, 98% at 9 min, 40% at 10 min, and 40% at 11 min. The total run time was 12 min per sample. Analytes were detected using a triple-quadrupole mass analyzer operating in positive ion mode (ESI^+^). For MS/MS analyses, norbelladine and norcraugsodine standards were characterized by the isolation of the parent mass in Q1, the specific fractionation of the parent molecules in a collision cell at a selected energy in q, and finally the scan of the characteristic ions fragments in Q2. The conditions of the MS/MS section were set to acquire in positive ion mode as follows: desolvation gas flow rate 708 L/hr., desolvation gaz temperature 400 °C, source temperature 120 °C, capillary voltage 1000 V, cone voltage 15 V, scan mass range from 100 to 265 + ESI and collision energy of 0–30 V. MassLynx software from Waters was used for data acquisition and processing.

Synthesized standard norbelladine (m/z 260) was analyzed using MS mode where the first two quadrupoles were set to radio frequency (RF) only and the third quadrupole scanned the mass range of 253–265 *m/z*. The obtained mass-to-charge (*m/z*) value and retention time were used to develop subsequent collision-induced dissociation (CID) experiments. Fragmentation spectrum was obtained using daughter mode at optimized collision energy (15 V) and the third quadrupole scanned the mass range of 50–275 *m/z.* Norbelladine eluted at 5.5 min with five significant [M + H]^+^ in daughter ion mode (260, 159, 138, 123, 121). Abundant fragment 138 m/z (using cone voltage 20 V and collision energy 10ev), 123 m/z (cone voltage 15 V and collision energy 20 eV) and 121 m/z (cone voltage 15 V and collision energy 20 eV) were optimized for qualifier and quantifier analysis in multi reaction monitoring mode (MRM) based on their signal intensity at an applied voltage (collision energy). MRM was used to measure the intensity of selected fragments to mark them as qualifier and quantifier ion. Norcraugsodine was also eluted at the similar retention time as norbelladine (5.5 min) with two significant [M + H]^+^ in daughter ion mode (258, 257 and 121). The fragment ion *m/z* 121 was detected in both standards and considered as the quantitative ion for analysis. CID fragmentation spectra for standards are available in Additional file 3. Qualitative ions for norbelladine and norcraugsodine were m/z 138 and m/z 157 respectively for validation. The MRM method thus developed was subsequently used to analyze the enzymatic essay samples. Quantification of standards and enzyme assay were performed using integration function of m/z 121 (quantifier daughter ion) on MassLynx software to obtain area under the chromatogram peak. Quantification of norbelladine in the enzyme essay sample was achieved by using the standard curve of norbelladine which has a linear equation of y = 31,264x + 2879 and a correlation coefficient (R^2^) of 0.999. Solutions of concentration between 0 and 10 ppm of norbelladine were prepared and each of them have been injected in triplicate in the LC-MS using the optimized MRM method for the quantitative daughter ion of norbelladine. The area under the chromatographic peaks for each solution was obtained by using the integration function of MassLynx software. The enzyme assay product was confirmed by comparison of LC-ESI-MS/MS data to standards.

## Additional files

Additional file 1: Three orthologs of NBS obtained from the *N. pseudonarcissus* transcriptome. (DOCX 81 kb)

Additional file 2: Sequences for NCS and PR-10 used in phylogenetic analysis (DOCX 18 kb)

Additional file 3: Fragmentation spectra obtained from LC-MS/MS analysis of standards norbelladine and norcraugsodine followed by a table listing the parameters used for LC-MS/MS analysis. (DOCX 76 kb)

Additional file 4: List of primer sequences used in this study. (DOCX 12 kb)

Additional file 5: Proton and carbon NMR spectral data of newly synthesized norcraugsodine and norbelladine. (DOCX 17385 kb)

## Abbreviations

3,4-DHBA: 3,4-dihydroxybenzaldehyde
AA: Amaryllidaceae Alkaloid
ESI: ElectroSpray Ionization
LC-MS: Liquid chromatography-Mass-spectrometry
m/z: mass to charge ratio
MRM: multiple reaction monitoringl
N4OMT: Norbelladine 4-O-methyltransferase
NBS: Norbelladine synthase
NCS: Norcloclaurine synthase
NR: Noroxomaritidine reductase
PR-10: Pathogenesis Related protein-10
