# Supplementary material for: Cloning and characterization of norbelladine synthase catalyzing the first committed reaction in Amaryllidaceae alkaloid biosynthesis
Source: BMC Plant Biol. 2018 Dec 7;18:338. doi: 10.1186/s12870-018-1570-4 (PMC6286614; doi:10.1186/s12870-018-1570-4)

**Additional file 1 :** Sequences for the three orthologs of NBS obtained from the *N. pseudonarcissus* transcriptome. The sequences are 97% identical.

| **Transcript ID** | **Gene length (bp)** | **ORF length (bp)** |
| --- | --- | --- |
| TR17354\|c0_g1_i1 | 593 | 492 |
| TR17354\|c0_g1_i2 | 575 | 480 |
| TR17354\|c0_g1_i3 | 583 | 483 |


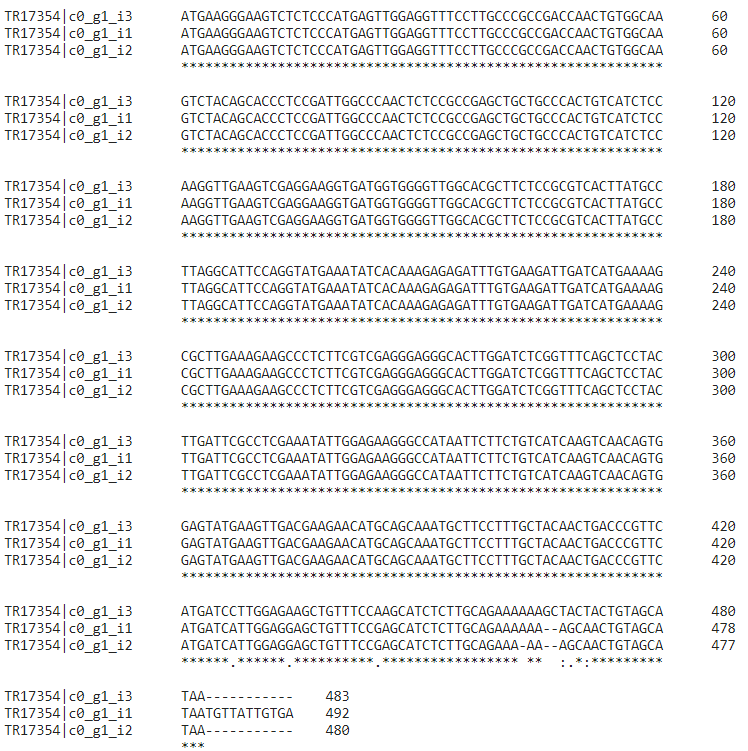

Supplement: Supplementary file 1 — Three orthologs of NBS obtained from the N. pseudonarcissus transcriptome. (DOCX 81 kb) [file 12870_2018_1570_MOESM1_ESM.docx]
