# Supplementary material for: Cloning and characterization of norbelladine synthase catalyzing the first committed reaction in Amaryllidaceae alkaloid biosynthesis
Source: BMC Plant Biol. 2018 Dec 7;18:338. doi: 10.1186/s12870-018-1570-4 (PMC6286614; doi:10.1186/s12870-018-1570-4)
Supplement: Supplementary file 2 — Sequences for NCS and PR-10 used in phylogenetic analysis (DOCX 18 kb) [file 12870_2018_1570_MOESM2_ESM.docx]

**Additional file 2 :** Sequences for NCS and PR-10 used in phylogenetic analysis

| **Short name** | **Species** | **Reference/Accession number** |
| --- | --- | --- |
| *AmNCS1* | *Argenome mexicana* | (Li, 2016) |
| *CjNCS2* | *Coptis japonica* | (Li, 2016) |
| *CmNCS1* | *Chelidonium majus* | (Li, 2016) |
| *DcPR10* | *Daucus carota* | (Sano, 2004) |
| *EcNCS1* | *Eschscholzia californica* | (Li, 2016) |
| *EcNCS2* | *Eschscholzia californica* | (Li, 2016) |
| *HoPR10* | *Hyacinthus orientalis* | (Liscombe, 2005) |
| *PmPR10* | *Pinus monticola* | (Liu, 2003) |
| *StPR10* | *Solanum tuberosum* | (Matton, 1989) |
| *TfNCS1* | *Thalictrum flavum* | (Li, 2016) |
| *TfNCS2* | *Thalictrum flavum* | (Li, 2016) |
| *TfNCS3* | *Thalictrum flavum* | (Li, 2016) |
| *TfNCS4* | *Thalictrum flavum* | (Li, 2016) |
| *TfNCS5* | *Thalictrum flavum* | (Li, 2016) |
| *PsNCS1* | *Papaver somniferum* | (Liscombe, MacLeod et al. 2005) |
| *PsNCS2* | *Papaver somniferum* | (Samanani and Facchini 2001) |
| *BpPR10* | *Betulla platyphylla* | (Li, 2016) |
| *BvPR10* | *Betulla verrucosa* | (Liu, 2006) |
| *ZmPR10* | *Zea mays* | (Schnable, Ware et al. 2009) |
| *HvPR10* | *Hordeum vulgare* | (Steiner-Lange, Fischer et al. 2003) |
| *OsPR10* | *Oryza sativa* | Unpublished (ACA50491) |
| *LrPR10* | *Lily regale* | Unpublished (ARX80137) |
| *SbPR10* | *Sorghum bicolor* | Unpublished (AAW83207) |
| *PsMLP* | *Papaver somniferum* | (Nessler and Burnett 1992) |
| *HYP-1* | *Hypericum perforatum* | (Michalska, Fernandes et al. 2010) |
| *NpNBS* | *Narcissus pseudonarcissus ‘King Alfred’* | This study |
