# Supplementary material for: Cloning and characterization of norbelladine synthase catalyzing the first committed reaction in Amaryllidaceae alkaloid biosynthesis
Source: BMC Plant Biol. 2018 Dec 7;18:338. doi: 10.1186/s12870-018-1570-4 (PMC6286614; doi:10.1186/s12870-018-1570-4)
Supplement: Supplementary file 3 — Fragmentation spectra obtained from LC-MS/MS analysis of standards norbelladine and norcraugsodine followed by a table listing the parameters used for LC-MS/MS analysis. (DOCX 76 kb) [file 12870_2018_1570_MOESM3_ESM.docx]

**Additional file 3:** Fragmentation spectra obtained from LC-MS/MS analysis of standards norbelladine and norcraugsodine followed by a table listing the parameters used for LC-MS/MS analysis.


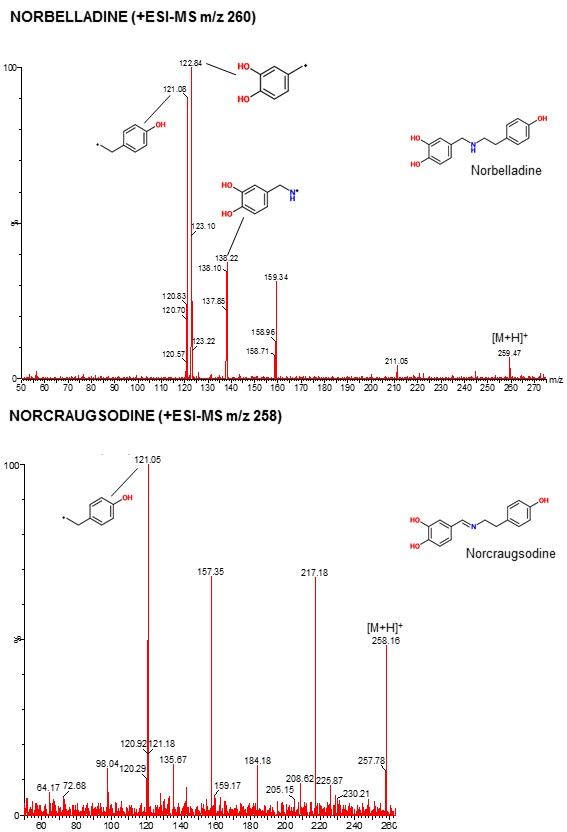


| **Compound** | **[M+H]^+^** | **RT**  **(min)** | **VC**  **(V)** | **CE daughters**  **(V)** | **Daughters, *m/z***  **(relative abundance)** | **Qualifier CE**  **(V)** | **Qualifier transition** | **Quantifier CE**  **(V)** | **Quantifier transition** |
| --- | --- | --- | --- | --- | --- | --- | --- | --- | --- |
| Norbelladine | 260 | 5.5 | 20 | 15 | 260 (8), 159 (30), 138 (39), 123 (100), 121 (90) | 15 | 260→138 | 20 | 260→121 |
| Norcraugsodine | 258 | 5.5 | 20 | 10 | 258 (50), 217 (68), 157 (68), 121 (100) | 15 | 258→157 | 20 | 258→121 |
