# Supplementary material for: Cloning and characterization of norbelladine synthase catalyzing the first committed reaction in Amaryllidaceae alkaloid biosynthesis
Source: BMC Plant Biol. 2018 Dec 7;18:338. doi: 10.1186/s12870-018-1570-4 (PMC6286614; doi:10.1186/s12870-018-1570-4)
Supplement: Supplementary file 4 — List of primer sequences used in this study. (DOCX 12 kb) [file 12870_2018_1570_MOESM4_ESM.docx]

| **Primer name** | **Primer Sequence** |
| --- | --- |
| *NpNBS*_GAT_F | 5’-GGGGACAAGTTTGTACAAAAAAGCAGGCT AGAAGGAGATATACATATGAAGGGAAGTCTCTCC-3’ |
| *NpNBS*_GAT_R | 5’-GGGGACCACTTTGTACAAGAAAGCTGGGTA CAATAACATTAT GCTACAG-3’ |
| *NpNBS*_qRT-PCR_F | 5’-GAGTTGGAGGTTTCC TTGC-3’ |
| *NpNBS*_qRT-PCR_F | 5’-CCACCATCACCTTCCTCG-3’ |
| *NpHIS*_ qRT-PCR_F | 5’-GTCTGCCCCAACAACTGGAGG-3’ |
| *NpHIS*_ qRT-PCR_F | 5’-GCTTCCTAATCAGTAGCTCG-3’ |

**Additional file 2 :** List of primer sequences used in this study.
