# Supplementary material for: Cloning and characterization of norbelladine synthase catalyzing the first committed reaction in Amaryllidaceae alkaloid biosynthesis
Source: BMC Plant Biol. 2018 Dec 7;18:338. doi: 10.1186/s12870-018-1570-4 (PMC6286614; doi:10.1186/s12870-018-1570-4)

**Additional file 5:** Proton and carbon NMR spectral data of newly synthesized norcraugsodine and norbelladine.

A) Norcraugsodine - Imine compound ^1^H NMR spectrum


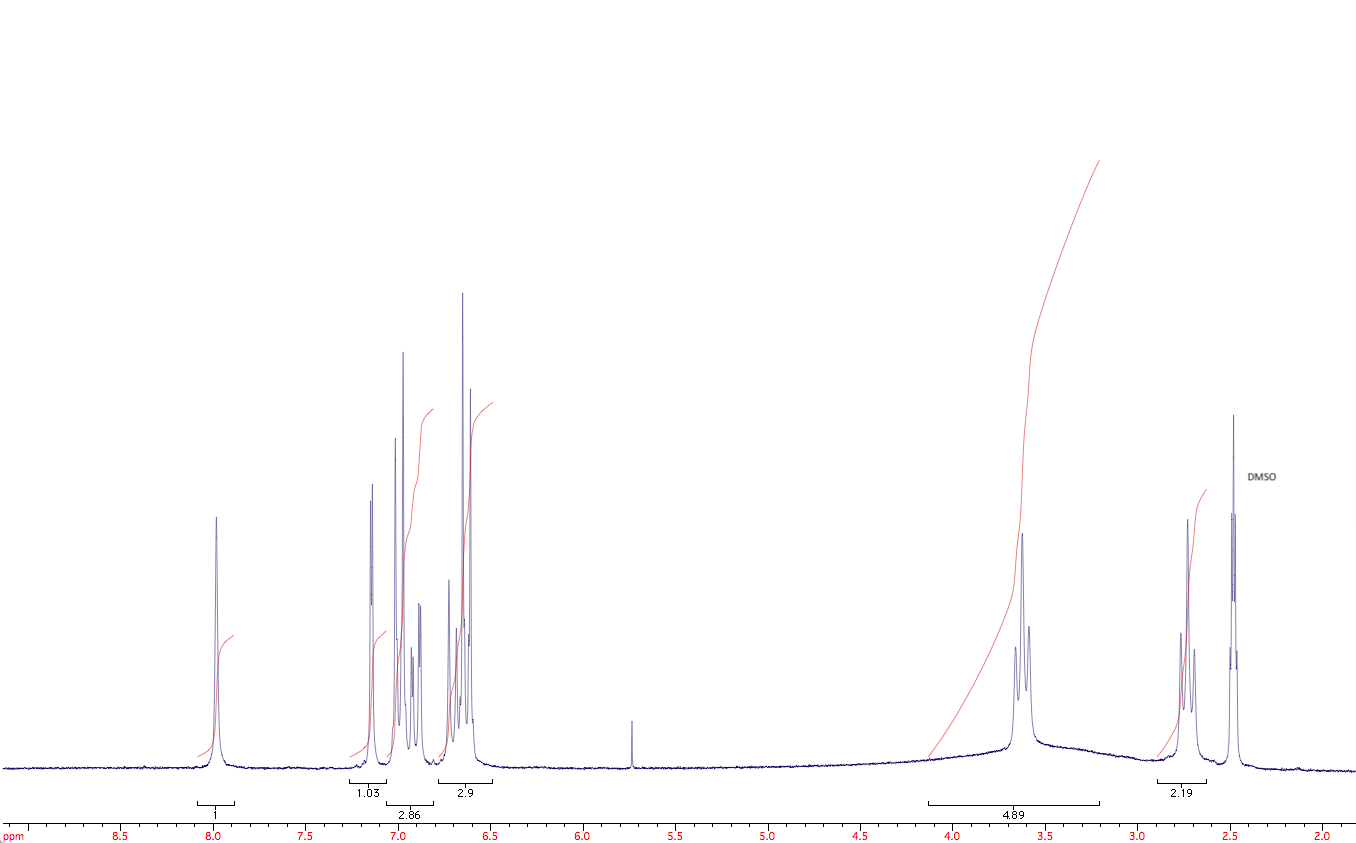


B) Norcraugsodine - Imine compound ^13^C NMR spectrum


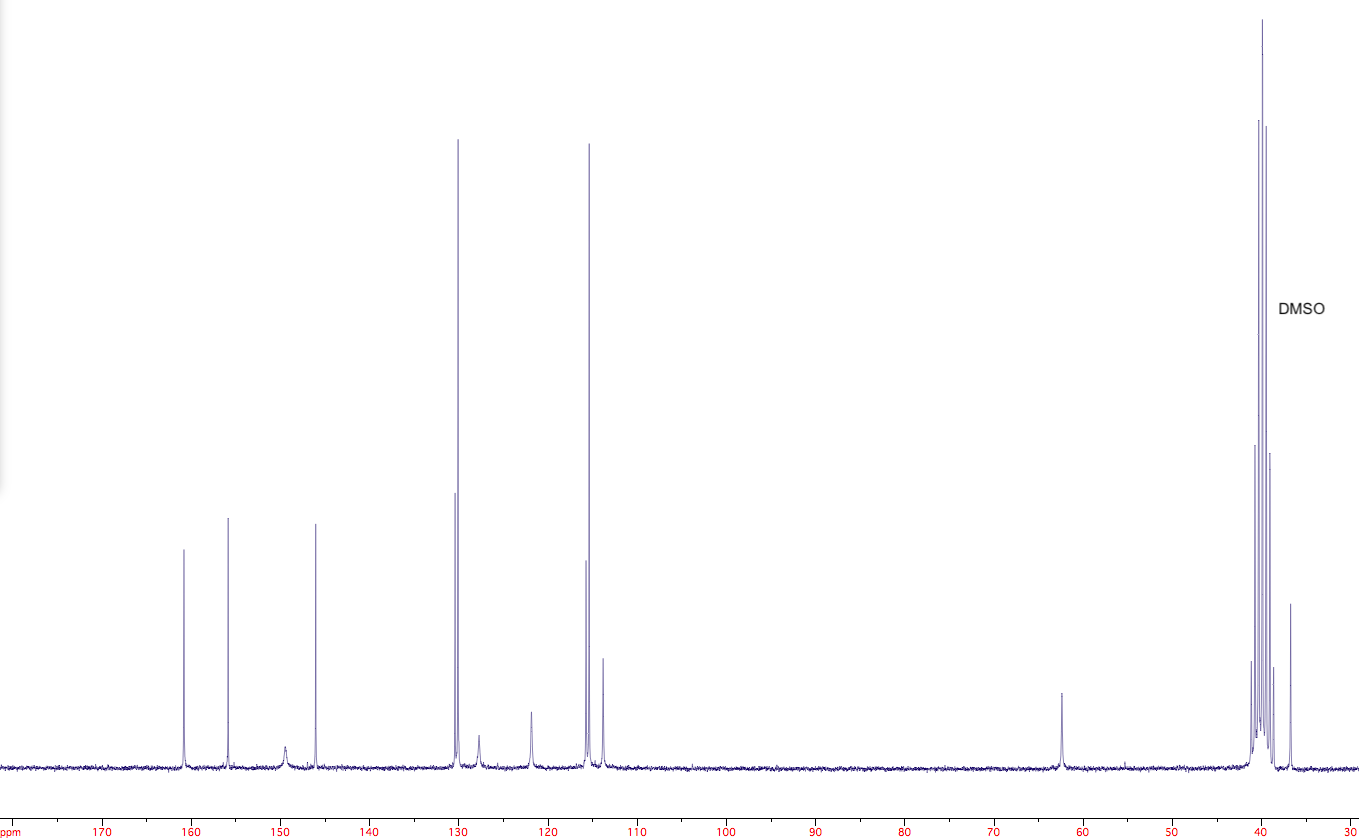


C) Norbelladine ^1^H NMR spectrum


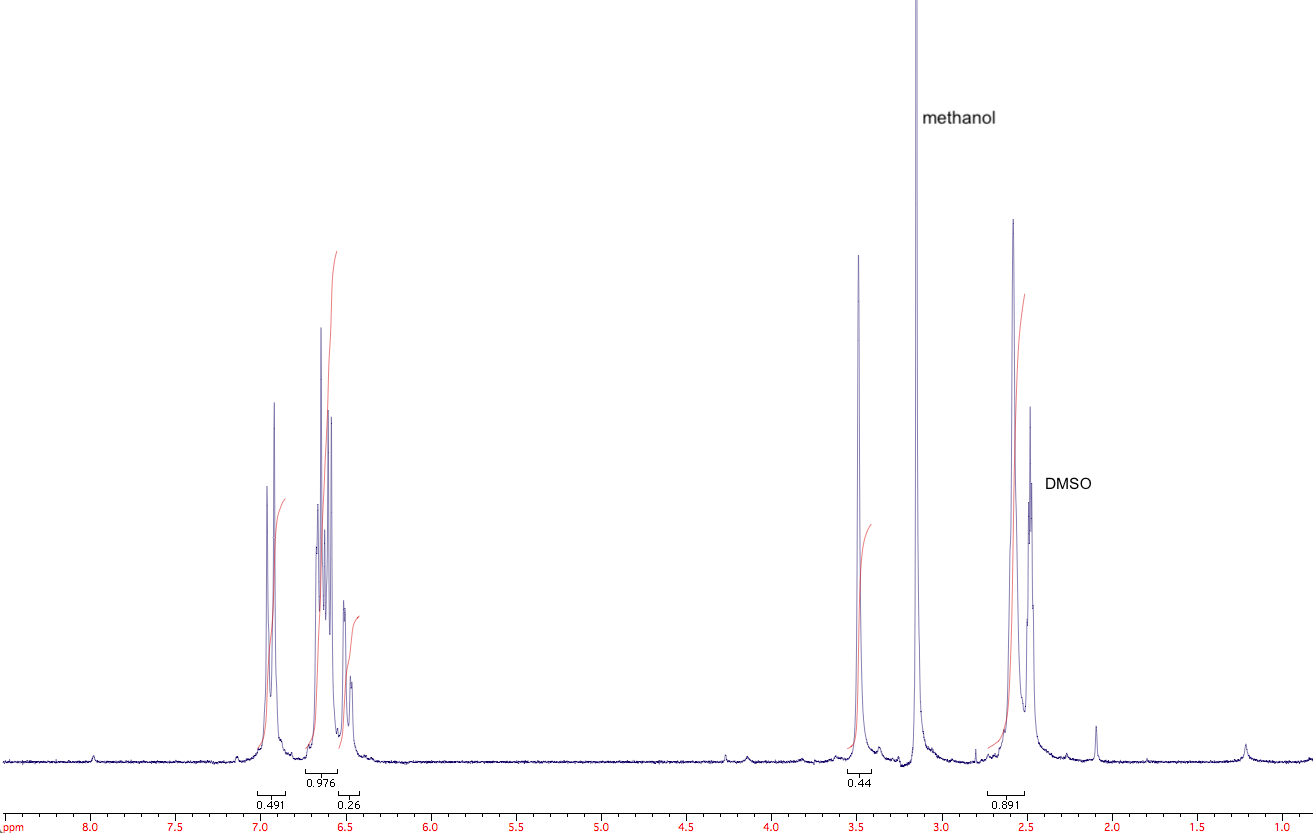


D) Norbelladine ^13^C NMR spectrum


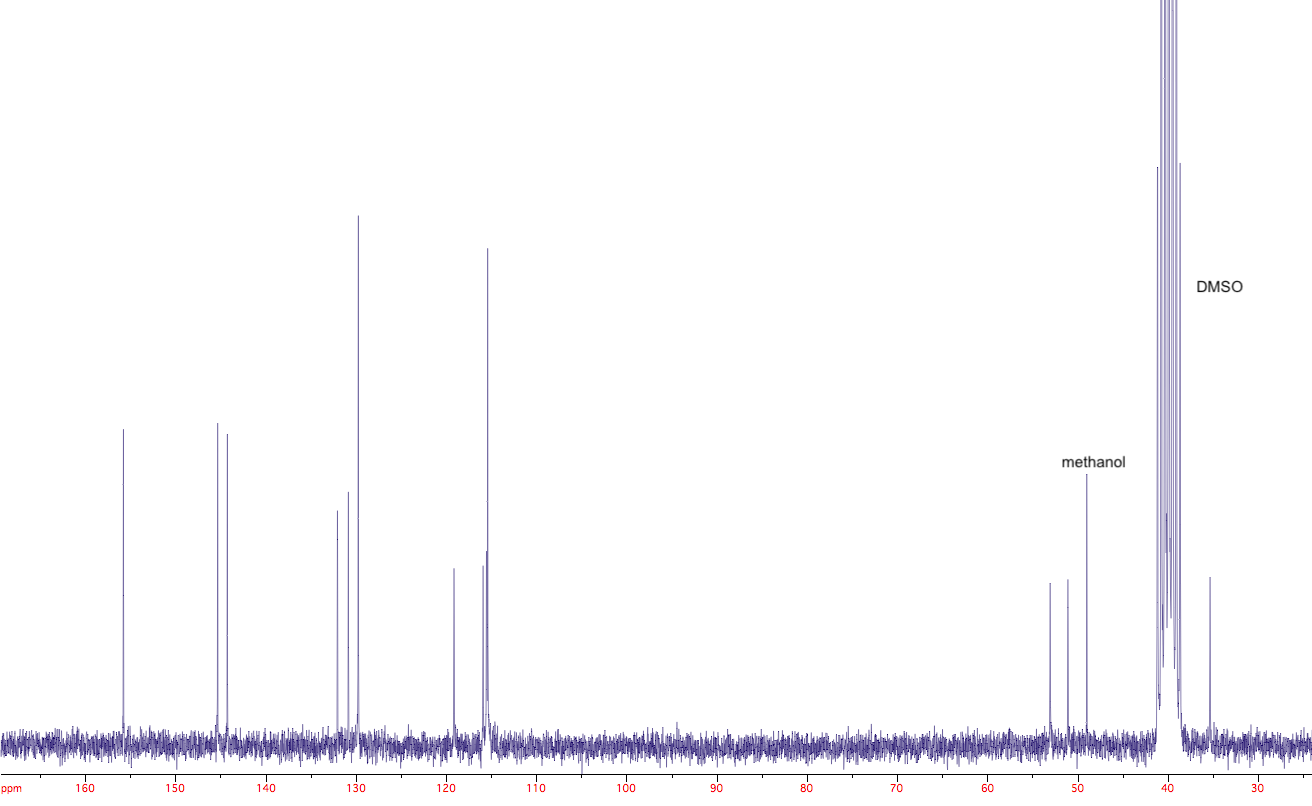

Supplement: Supplementary file 5 — Proton and carbon NMR spectral data of newly synthesized norcraugsodine and norbelladine. (DOCX 17385 kb) [file 12870_2018_1570_MOESM5_ESM.docx]
